# Atypical Lesions in Virchow-Robin Spaces: A Case Report

**DOI:** 10.7759/cureus.77445

**Published:** 2025-01-14

**Authors:** Anderson M Pereira da Silva, Vitoria P Junges, Virna F Muniz, Raissa C Leite Lucio Silva, Caio Borges S Guimarães

**Affiliations:** 1 Pharmacology, Universidade Federal do Vale do São Francisco, Petrolina, BRA; 2 Neurological Surgery, Federal University of Roraima, Boa Vista, BRA; 3 Medical School, Federal University of Roraima, Boa Vista, BRA; 4 Cardiology, Father Albino Hospital, Catanduva, BRA; 5 Anesthesiology, Universidad Abierta Interamericana, Buenos Aires, ARG

**Keywords:** brain lesions, differential diagnosis, surgical case reports, tumefactive lesions, virchow-robin spaces

## Abstract

The differentiation between benign and malignant brain lesions remains a fundamental challenge in modern neuroimaging. This case highlights a rare presentation of ectatic Virchow-Robin spaces (VRS), which mimicked tumefactive brain lesions and required a comprehensive diagnostic evaluation to exclude neoplastic, infectious, and inflammatory processes. A 37-year-old female presented with progressive headache, cognitive impairment, and facial pain. Magnetic resonance imaging revealed a multiloculated subcortical cystic lesion, initially raising suspicions of a dysembryoplastic neuroepithelial tumor or neurocysticercosis. Advanced imaging modalities, including MR spectroscopy and MR angiography, played a crucial role in confirming the benign nature of the lesion. This report underscores the critical role of a multidisciplinary diagnostic approach, integrating clinical presentation, imaging characteristics, and laboratory findings, to address the diagnostic overlap between benign dilated VRS and pathological conditions such as tumors or infections. Additionally, the case emphasizes the importance of recognizing atypical presentations of VRS to avoid unnecessary interventions and to guide patient-centered management.

## Introduction

Virchow-Robin spaces (VRS), also known as perivascular spaces, are anatomical structures in the brain that accompany blood vessels as they penetrate the cerebral parenchyma. These spaces, filled with cerebrospinal fluid, play an important role in cerebral homeostasis, acting as drainage pathways and participating in immunological and metabolic processes. While generally considered benign and asymptomatic structures, alterations in these spaces can be associated with various pathological conditions, including inflammatory, neoplastic, and demyelinating processes. The presence of atypical lesions in these spaces poses a significant diagnostic challenge, given the overlap of clinical and radiological features among different etiologies [1-4].

The differential diagnosis of lesions in the VRS is particularly complex, as these structures may be involved or enlarged by a wide range of pathologies. Inflammatory conditions, such as encephalitis or autoimmune diseases, may mimic neoplastic or demyelinating alterations, while brain tumors or demyelinating processes, such as multiple sclerosis, may exhibit features that overlap with inflammatory lesions. This complexity requires a multidisciplinary approach, combining clinical data, advanced imaging studies, and, in many cases, histopathological analyses to achieve an accurate diagnosis [1,5-7].

In this case report, we discuss an atypical presentation of a lesion in the VRS, highlighting the diagnostic and therapeutic challenges encountered. The complexity of differentiating between inflammatory, neoplastic, and demyelinating etiologies is explored, aiming to contribute to diagnostic clarification and emphasize the importance of a careful and systematic approach to the management of brain lesions. This case illustrates the need for a high level of clinical suspicion and the use of complementary diagnostic tools to guide appropriate therapeutic decisions and improve patient outcomes.

## Case presentation

Case report

This case report was prepared per the 2013 CARE Checklist (CAse REport Statement and Checklist), ensuring a comprehensive and transparent presentation of the findings [8,9]. 

Patient information

**Figure 1:**
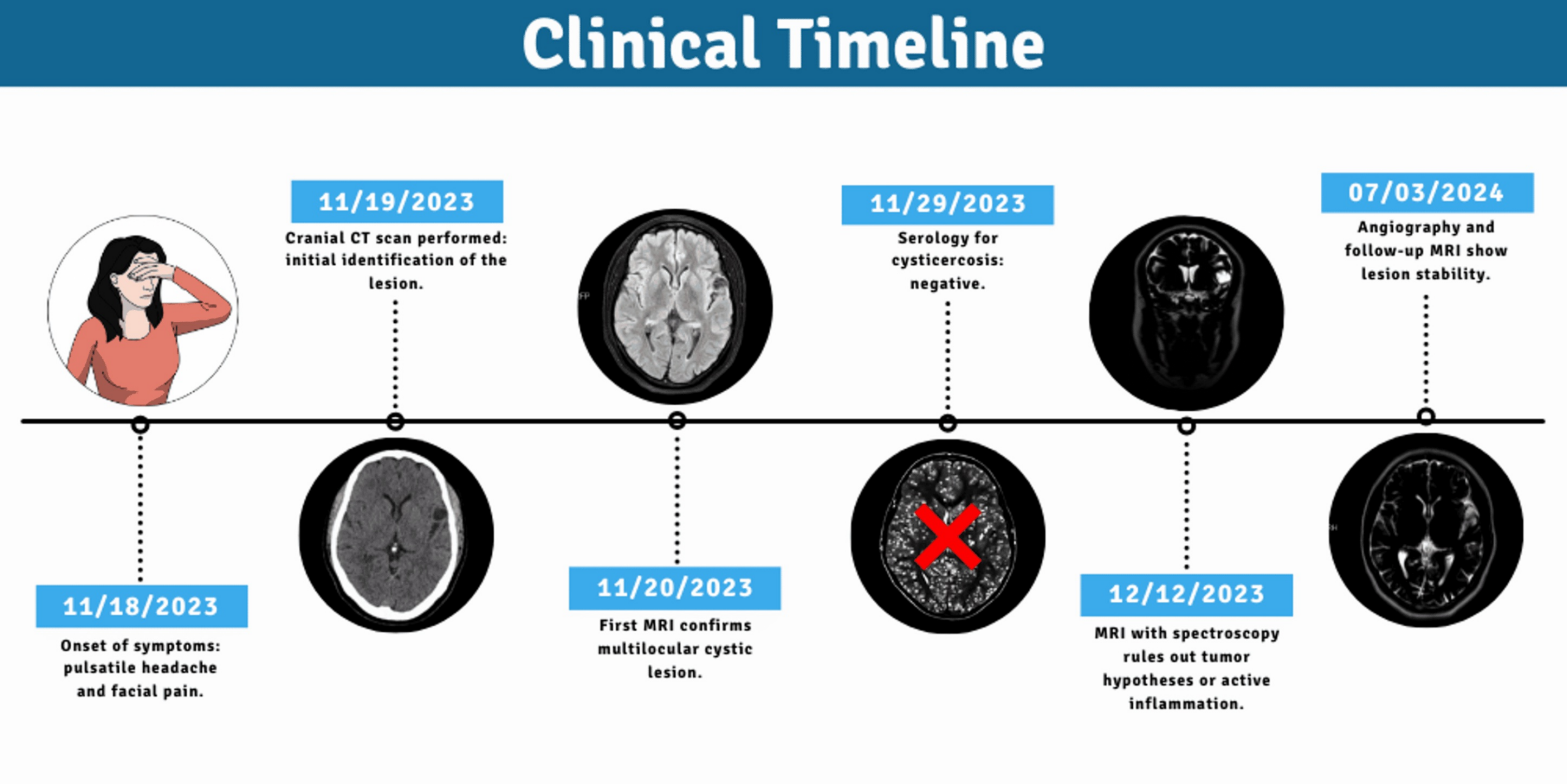
Clinical timeline of the case report, illustrating key diagnostic and follow-up milestones. Flowchart created according to 2013 CARE guidelines. Abbreviations: CT, computed tomography; MRI, magnetic resonance imaging; MRA, magnetic resonance angiography; EEG, electroencephalogram Created by the authors using mindthegraph.com.

The patient, a 37-year-old female, has a significant clinical history. She was diagnosed with Crohn's Disease (CD) in 2010 and has been on ustekinumab (UST) 90 mg since September 2018 as maintenance immunomodulatory therapy. In January 2019, she was diagnosed with pulmonary tuberculosis (PT), which was treated with a complete six-month pharmacological regimen (rifampin, isoniazid, pyrazinamide, and ethambutol). As a consequence of CD and TB, the patient developed immunosuppression, a factor that may influence the presentation and progression of complex neurological and systemic clinical conditions. A detailed timeline of events related to the patient's management is provided in Figure 1.

Clinical presentation

**Figure 2:**
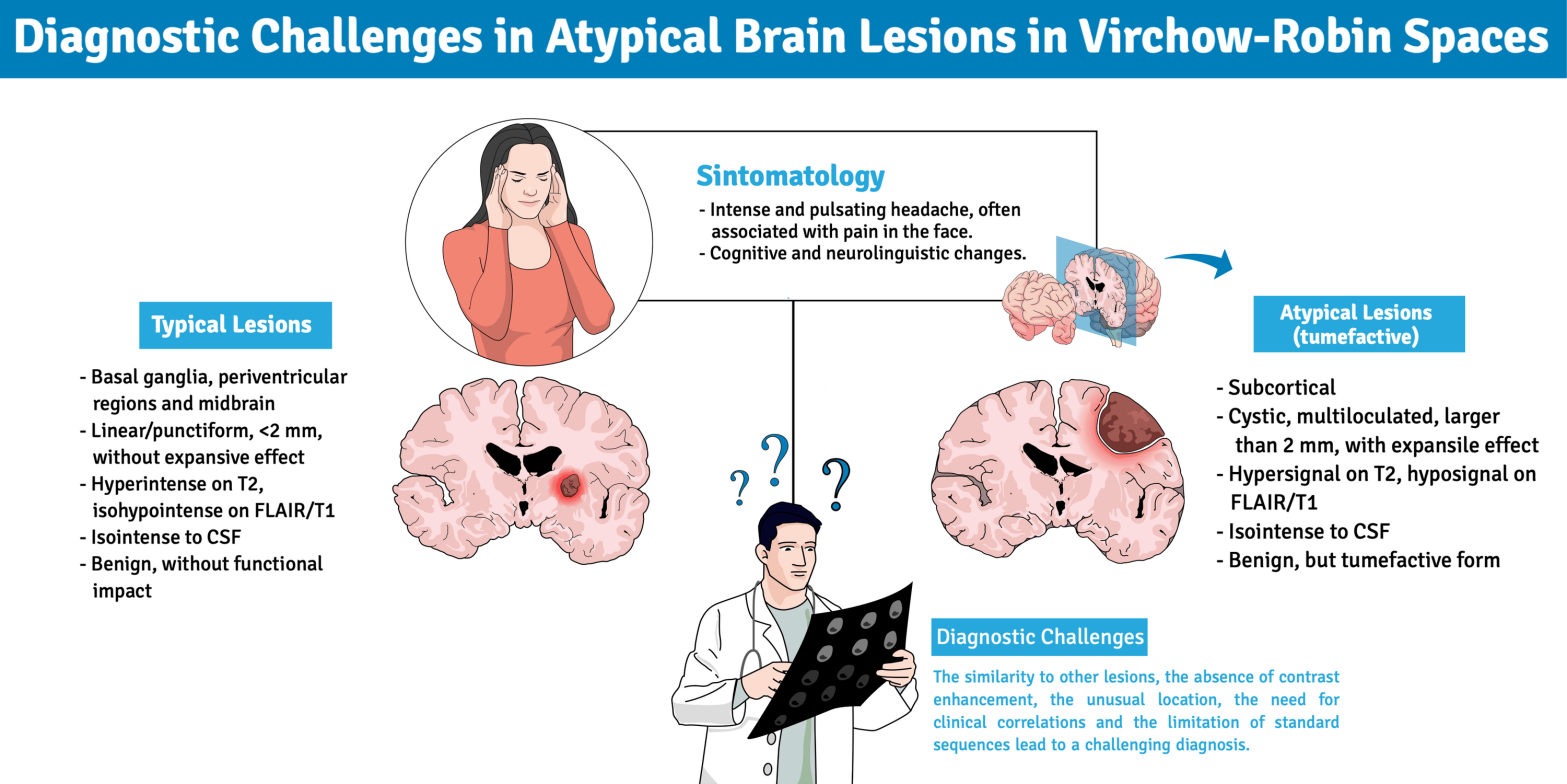
Comparison of typical and atypical presentations of brain lesions within Virchow-Robin spaces Abbreviations: CSF, cerebrospinal fluid, FLAIR, fluid-attenuated inversion recovery; T2, T2-weighted imaging; T1, T1-weighted imaging Created by the authors using mindthegraph.com

The patient presented with a sudden onset of symptoms, including intense, pulsating frontal headache accompanied by facial pain. Clinical progression was characterized by difficulties in writing, speech articulation (dysarthria and receptive and expressive aphasia), including letter substitutions (e.g., replacing "D" with "B"), and progressive memory impairment. The persistence and worsening of symptoms prompted the patient to seek emergency medical evaluation. Figure 2 illustrates the main challenges in the differential diagnosis of lesions in VRS, highlighting both typical and atypical features identified in this case.

Diagnostic assessment

**Figure 3:**
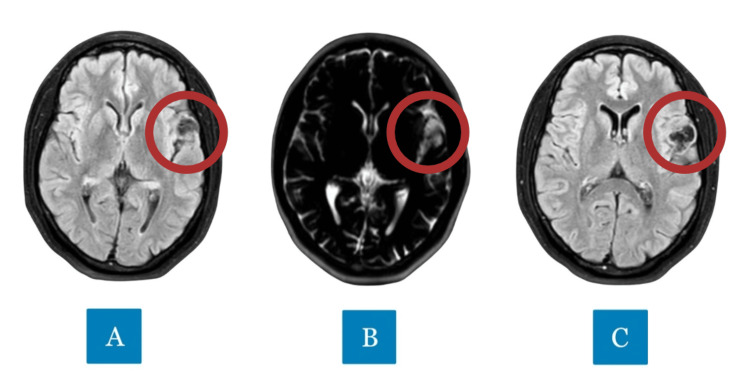
A (axial T2-weighted): Axial image showing hyperintensity characteristic of multiloculated subcortical cystic lesions; B (axial T1-weighted): axial image showing hypointensity, suggesting a lesion with cystic or cerebrospinal fluid content; C (FLAIR): axial image demonstrating hypointensity, indicating iso-intensity to cerebrospinal fluid.

In the initial evaluation, imaging studies were fundamental for the diagnostic investigation. Magnetic resonance imaging (MRI) of the brain revealed a multiloculated subcortical cystic lesion with a mild expansive effect, located in the left frontotemporoparietal operculum. The lesion measured approximately 2.9 × 1.9 × 1.6 cm. Signal characteristics showed T2 hyperintensity, FLAIR/T1 hypointensity, and an absence of contrast enhancement or diffusion restriction, findings consistent with the hypothesis of ectatic VRS in a tumefactive presentation (Figure 3). In Figure 3, A (axial T2-weighted) shows hyperintensity, characteristic of multiloculated cystic lesions compatible with dilated VRS. In B (axial T1-weighted), hypointensity is observed, suggesting a lesion with cystic or cerebrospinal fluid content, without expansive characteristics. In C (FLAIR), hypointensity is evident, indicating isointensity to cerebrospinal fluid, consistent with benign alterations associated with VRS. These findings, combined with a negative serological test for neurocysticercosis and a normal electroencephalogram (EEG), supported the diagnosis of a benign cystic lesion, most likely associated with ectatic VRS.

Therapeutic interventions

Management involved regular follow-up imaging to monitor lesion stability. The benign nature of the lesion negated the need for invasive procedures, and conservative management was deemed sufficient.

Differential diagnoses

The diagnostic evaluation considered several hypotheses based on clinical and radiological findings. Dysembryoplastic neuroepithelial tumor (DNET) was initially suggested due to some partially compatible radiological features. However, the absence of metabolic alterations on MR spectroscopy and the lack of contrast enhancement ruled out this possibility. Neurocysticercosis was also considered, particularly due to the initial presentation and the cystic pattern of the lesion. However, negative serology for cysticercosis, combined with the absence of typical features such as ring-enhancing cysts, excluded this diagnosis.

A congenital malformation, specifically an anatomical variant of the perivascular spaces, was included as a diagnostic hypothesis, aligning with the final diagnosis of ectatic VRS. This rare condition exhibited multiloculated cystic features on MRI, corroborated by imaging findings and the radiological stability observed during follow-up.

## Discussion

The presented case highlights the diagnostic challenges associated with dilated VRS, a condition that, although generally benign, can mimic neoplastic, infectious, or inflammatory processes. VRS, also known as perivascular spaces, surround cerebral vessels and play essential roles in fluid exchange between the blood, cerebrospinal fluid, and the brain interstitium [10,11]. Typically measuring less than 2 mm in diameter, these spaces can dilate, reaching larger dimensions and becoming macroscopically visible on MRI [12]. Their atypical and tumefactive presentation, as observed in this case, poses significant diagnostic challenges. Studies indicate that dilated VRS is associated with various clinical conditions, including small vessel disease, hypertension, aging, and neuroinflammatory disorders such as multiple sclerosis [13,14]. In multiple sclerosis patients, for instance, an increased VRS burden has been correlated with inflammatory changes and possibly with the emergence of contrast-enhancing lesions [11]. However, the clinical significance of dilated VRS, particularly in atypical presentations, remains widely debated.

In this case, the tumefactive presentation of VRS raised challenging diagnostic questions, requiring the exclusion of hypotheses such as neural tumors, neurocysticercosis, and congenital malformations. MR spectroscopy demonstrated normal metabolic patterns, with no elevations in choline or other markers indicative of malignancy, reinforcing the benign nature of the dilated VRS. Additionally, radiological findings, including T2 hyperintensity and FLAIR/T1 hypointensity, mimicked processes such as DNET, but the absence of contrast enhancement and metabolic changes ruled out this possibility [15,16].

Beyond their role as incidental findings, dilated VRS have been associated with changes in cerebral fluid dynamics and the glymphatic system, with potential implications for neurodegenerative and inflammatory conditions. Evidence also suggests a possible link between VRS and systemic inflammatory processes, as demonstrated by the association between circulating inflammatory markers and MRI-visible VRS [10,17].

The diagnostic process in this case faced significant challenges, particularly in distinguishing between inflammatory, infectious, and neoplastic processes. These overlapping clinical and radiological features necessitated a comprehensive approach, integrating advanced imaging modalities. MR spectroscopy was crucial for ruling out neoplastic and infectious processes by analyzing metabolic patterns.

## Conclusions

This case highlights the importance of a systematic, multidisciplinary approach to diagnosing dilated VRS. In our case, the accurate identification of VRS as a benign etiology avoided unnecessary interventions and emphasized the role of advanced imaging techniques, such as MR spectroscopy, in confirming the diagnosis. Additionally, the tumefactive presentation of VRS mimicked neoplastic and infectious lesions, underscoring the diagnostic challenges associated with these atypical forms. The proposed term "Virchoma" provides a framework to differentiate such cases. Further research is needed to clarify the clinical significance of dilated VRS, particularly in atypical presentations, to enhance diagnostic precision and patient care.
